# Myeloperoxidase Deficiency: The Secret Under the Flag of Unstained Cell

**DOI:** 10.4274/Tjh.2012.0012

**Published:** 2013-06-05

**Authors:** Türkan Patıroğlu, Hatice Eke Güngör, Julie Sawalle Belohradsky, Ekrem Ünal, Christoph Klein

**Affiliations:** 1 Erciyes University, School of Medicine, Department of Pediatrics, Division of Pediatric Hematology and Oncology, Kayseri, Turkey; 2 Erciyes University, School of Medicine, Department of Pediatrics, Division of Pediatric Allergy and Immunology, Kayseri, Turkey; 3 University Children’s Hospital, Molecular Genetic Diagnostics, Infektionsimmunologisches Labor, Munich, Germany; 4 University Children’s Hospital, Department of Pediatrics, Division of Pediatric Hematology and Oncology, Munich, Germany

**Keywords:** Leucocyte function disorders, Myeloperoxidase, Neutrophil

Myeloperoxidase (MPO) deficiency is one of the most common inherited phagocyte defects, but it is rarely associated with clinical symptoms [1]. MPO, which is abundant in azurophilic granules of neutrophils and in the lysosomes of monocytes, plays a key role in amplifying the toxicity of hydrogen peroxide generated by the respiratory burst [1,2]. The diagnosis of MPO deficiency was rare before 1979; currently, the diagnosis is easily made due to the widespread use of automated flow cytochemical analysis in clinical hematology laboratories for enumerating peripheral blood neutrophils with peroxidase activity [2]. 

The MPO gene is encoded on chromosome 17q23 [3]. Primary deficiency of MPO is inherited as an autosomal recessive disorder. A secondary form of MPO deficiency has been described in various disorders, including lead poisoning, severe infections, neuronal lipofuscinosis, obstructive jaundice, diabetes mellitus, and such disseminated cancers as acute and chronic myeloid leukemia, myelodysplastic syndrome, and Hodgkin’s lymphoma [1,2,4]. MPO-deficient neutrophils are markedly less efficient at killing Candida albicans and Aspergillus hyphae [1]. Although MPO is involved in killing certain microorganisms, to date, no particular susceptibility to persistent or severe infections has been noted in the vast majority of MPO-deficient patients [1,2]. Recurrent Candida infection has been observed predominantly in MPO-deficient patients that also have diabetes mellitus. In addition to infectious manifestations, the relationship between congenital MPO deficiency and the occurrence of malignancy remains controversial [1,2]. There is no specific treatment for MPO deficiency; however, in symptomatic patients antifungal treatment is crucial. 

Herein we present a 17-year-old male that presented to our hematology department with neutropenia. Anamnesis showed that the patient did not have any recurrent or severe infection, but had been receiving benzathine penicillin prophylaxis due to acute rheumatic fever for years. Physical examination showed the following: weight: 58 kg (3-10p); height: 169 cm (10-25p); 1-2-degree/6 systolic murmur in the mesocardial region; the remainder of the examination was unremarkable. 

Complete blood count results were as follows: hemoglobin: 13.1 g/dL; leucocyte count: 6140/mm^3^; platelet count: 503,000/mm^3^. The formulation of the leucocytes was interesting, with an absolute neutrophil count of 20/mm^3^ and a high absolute unstained cell count of 3020/mm^3^ (nearly 50%). Although the absolute neutrophil count was 20/mm^3^, peripheral blood smear showed normal formulation of leucocytes (49% neutrophils, 47% lymphocytes, and 4% monocytes). The inconsistency between the absolute neutrophil count and the peripheral blood smear suggested the possibility of MPO deficiency, and we therefore performed cytochemical staining of peroxidase and flow cytometry analysis. Cytochemical staining of peroxidase was negative, as compared to the control (Figure 1a, 1b), and flow cytometry analysis showed a dramatic decrease in MPO(+) neutrophils (Figure 2). In addition, we identified a compound heterozygous MPO gene mutation [c.1705C> T];[c.2031-2A>C]. The patient was followed-up for 12 months after his initial examination, during which time he was asymptomatic and healthy. In conclusion, we think that MPO deficiency must be considered in patients with incompatibility of neutropenia between smear and counter, especially when the flag of unstained cell is remarked. 

## CONFLICT OF INTEREST STATEMENT

The authors of this paper have no conflicts of interest, including specific financial interests, relationships, and/or affiliations, relevant to the subject matter or materials included.

## Figures and Tables

**Figure 1:**
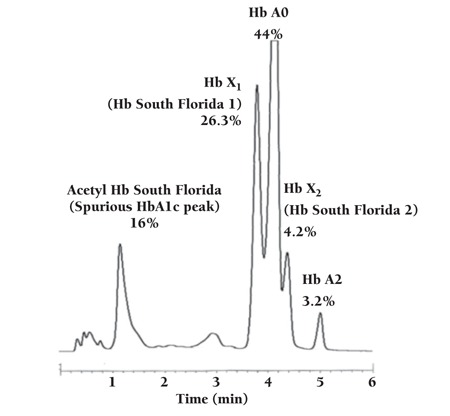
The cytochemical staining of the neutrophil from patient (a) shows negativity when compared to the control (b).

**Figure 2:**
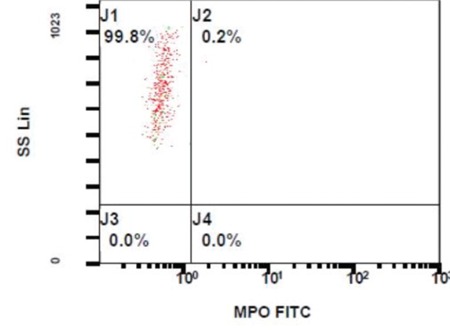
The flow cytometry analysis showed reduced positivity of myeloperoxidase at the gate of neutrophils.
